# Clostridium perfringens-Induced Emphysematous Pyelonephritis: A Diagnostic Challenge With Negative Urine Culture

**DOI:** 10.7759/cureus.88227

**Published:** 2025-07-18

**Authors:** Chisato Nakajima, Masahiro Kashiura, Shiho Togo, Haruka Taira, Hiroyuki Tamura, Hideto Yasuda, Takashi Moriya

**Affiliations:** 1 Department of Emergency and Critical Care Medicine, Jichi Medical University Saitama Medical Center, Saitama, JPN

**Keywords:** clostridium perfringens, emphysematous pyelonephritis, hemolysis, sepsis, urinary tract infections

## Abstract

*Clostridium perfringens* is rarely identified as a causative agent of urinary tract infections, and its anaerobic nature creates diagnostic challenges in clinical settings. We report the case of a 59-year-old woman with diabetes mellitus and stage IVA cervical cancer who presented with fever and dyspnea with acute onset, two days following ureteral stent placement. Clinical examination revealed costovertebral angle tenderness and dark red urine. Laboratory findings demonstrated hemolysis, renal dysfunction, and elevated inflammatory markers. Urinary Gram stain revealed Gram-positive rods, and computed tomography demonstrated gas in the right renal pelvis. Blood cultures yielded *C. perfringens*, yet urine cultures remained negative. The patient developed circulatory collapse requiring intensive care management. Following antimicrobial therapy and stent replacement, she stabilized and was discharged from the intensive care unit after six days.

This case highlights *C. perfringens* as a potential cause of healthcare-associated urinary tract infections in high-risk patients. The occurrence of negative urine cultures despite active infection underscores the necessity for anaerobic culture techniques, while intravascular hemolysis provides valuable diagnostic clues. Clinicians should consider anaerobic pathogens in urinary tract infections with unusual presentations, particularly in patients with malignancy, diabetes, or recent urological interventions.

## Introduction

Emphysematous pyelonephritis (EPN) is a severe, necrotizing infection characterized by gas formation within the renal system. This potentially life-threatening condition primarily affects patients with diabetes mellitus and urinary tract obstruction, with mortality rates ranging from 7% to 75% [1]. While *Escherichia coli* is the predominant causative pathogen (60-70% of cases), anaerobic bacteria such as *Clostridium perfringens* are rarely isolated from EPN cases [1,2].

*C. perfringens* is a Gram-positive, anaerobic bacillus that produces multiple toxins, notably alpha-toxin, which causes hemolysis through phospholipase C activity [3]. *C. perfringens* bacteremia carries significant mortality (27-44%), requiring prompt identification and treatment [4]. Diagnosing* C. perfringens* urinary tract infections (UTIs) is challenging due to its obligate anaerobic nature, limiting growth under routine aerobic urine culture conditions. Additionally, *Clostridium* species can undergo rapid decolorization during Gram staining, potentially leading to misidentification [5].

We present a case of healthcare-associated EPN caused by *C. perfringens* with negative routine urine cultures but positive blood cultures and characteristic intravascular hemolysis. This case emphasizes the importance of considering anaerobic pathogens in UTIs with unusual presentations, particularly in high-risk patients with recent urological interventions.

## Case presentation

A 59-year-old woman presented to our hospital with a 12-hour history of fever and dyspnea. Her medical history included diabetes mellitus and stage IVA cervical cancer. She was receiving chemotherapy and had a right ureteral stent inserted due to ureteral invasion from cervical cancer two days before admission.

**Figure 1:**
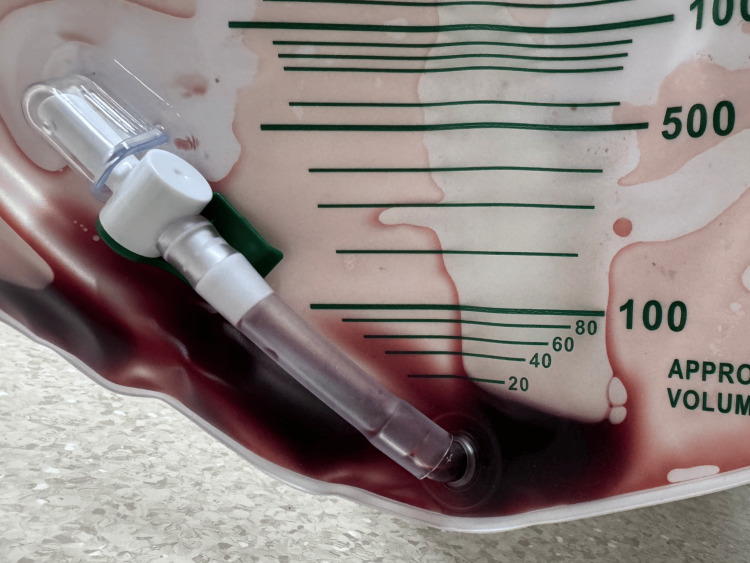
The patient's urine on admission Urine specimen shows dark red coloration suggestive of intravascular hemolysis.

Upon arrival, her vital signs were as follows: Glasgow Coma Scale score was E3V5M6, respiratory rate 33 breaths/min, heart rate 154 beats/min, blood pressure 150/77 mmHg, oxygen saturation 99% (ambient air), and body temperature 38.4℃. Physical examination revealed conjunctival pallor, jaundice, and costovertebral angle tenderness. The patient's urine demonstrated dark red discoloration consistent with hematuria or hemoglobinuria (Figure 1).

Laboratory findings were as follows: lactate 8.3 mmol/L, total bilirubin 6.45 mg/dL, direct bilirubin 3.86 mg/dL, aspartate aminotransferase 30 U/L, alanine aminotransferase 14 U/L, lactate dehydrogenase 787 U/L, potassium 6.5 mmol/L, creatine kinase 199 U/L, C-reactive protein 26.96 mg/dL, creatinine 2.62 mg/dL, white blood cell count 5.63×10^3^/μL, hemoglobin 6.9 g/dL, mean corpuscular volume 108.6 fL, platelets 3.7×10⁴/μL, prothrombin time-international normalized ratio 2.31, and D-dimer 10.2 μg/mL (Table 1). The peripheral blood smear revealed significant red blood cell abnormalities, including polychromasia and the presence of nucleated red blood cells. Additionally, neutrophils demonstrated marked toxic changes, such as toxic granulation, Döhle bodies, and vacuolation. Collectively, these findings are consistent with a leukoerythroblastic reaction, suggesting a severe underlying condition such as sepsis or massive hemolysis.

**Table 1:** The laboratory data at presentation TP: total protein; Alb: albumin; T-Bil: total bilirubin; D-Bil: direct bilirubin; AST: aspartate aminotransferase; ALT: alanine aminotransferase; LDH: lactate dehydrogenase; CK: creatine kinase; ALP: alkaline phosphatase; γ-GTP: gamma-glutamyl transpeptidase; CRP: C-reactive protein; Na: sodium; K: potassium; Cl: chlorine; Ca: calcium; P: phosphorus; Mg: magnesium; BUN: blood urea nitrogen; Cr: creatinine; WBC: white blood cell; RBC: red blood cell; Hb: hemoglobin; Ht: hematocrit; PLT: platelets; Fib: fibrin degradation products; PT-INR: prothrombin time-international normalized ratio; APTT: activated partial thromboplastin time; Lac: lactate

Parameters	Patient's value	Unit	Reference range
TP	5.9	g/dL	6.6-8.1
Alb	2.4	g/dL	4.1-5.1
T-Bil	6.45	mg/dL	0.4-1.5
D-Bil	3.86	mg/dL	0.1-0.2
AST	30	U/L	13-30
ALT	14	U/L	7-23
LDH	787	U/L	124-222
CK	199	U/L	41-153
ALP	334	U/L	38-113
γ-GTP	55	U/L	9-32
CRP	26.96	mg/dL	0-0.14
Na	140	mmol/L	138-145
K	6.5	mmol/L	3.6-4.8
Cl	108	mmol/L	100-110
Ca	7.6	mg/dL	8.4-10.1
P	3.8	mg/dL	2.7-4.6
Mg	1.3	mg/dL	1.7-2.5
BUN	55	mg/dL	8-20
Cr	2.62	mg/dL	0.46-0.79
WBC	5.63	×1000/μL	3.5-9.1
RBC	221	×10000/μL	376-500
Hb	6.9	g/dL	11.3-15.2
Ht	24	%	33.4-44.9
PLT	3.7	×10000/μL	13-36.9
Fib	545	mg/dL	200-400
PT-INR	2.31	-	0.9-1.2
APTT	40	sec	28.5-40.9
D-dimer	10.2	μg/mL	0-1
Lac	8.3	mmol/L	-

**Figure 2:**
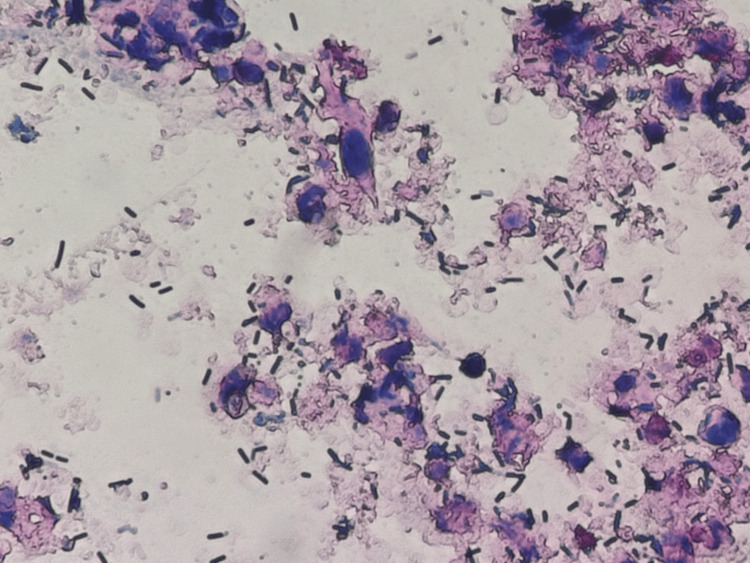
The Gram stain findings of urine Gram stain of urine demonstrates Gram-positive rods and phagocytosis by neutrophils.

**Figure 3:**
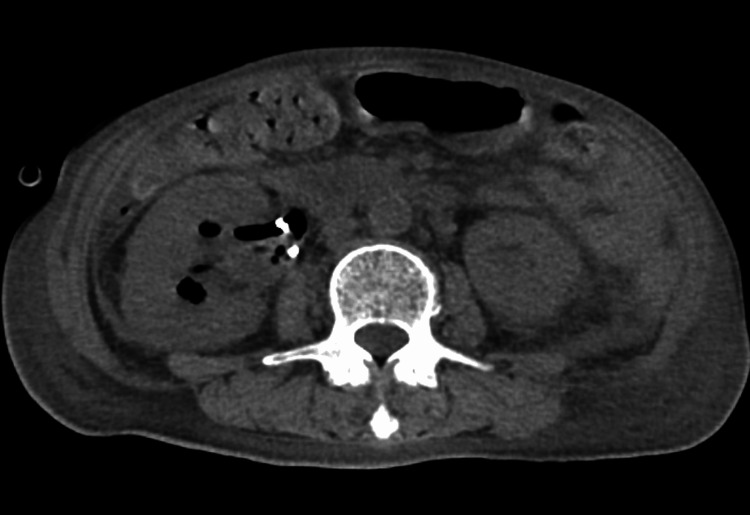
Computed tomography findings on admission Computed tomography shows an enlarged right kidney with gas formation in the renal pelvis and calyces, consistent with emphysematous pyelonephritis. A ureteral stent is in situ.

These findings indicated hemolysis, renal dysfunction, elevated inflammatory markers, anemia, and coagulopathy. Urinalysis revealed no red blood cells but positive occult blood, suggesting hemoglobinuria. Urinary Gram staining revealed Gram-positive rods (GPR) (Figure 2). Computed tomography revealed gas in the right renal pelvis and calyces, along with the ureteral stent (Figure 3). Ceftriaxone was initiated based on a diagnosis of EPN and sepsis.

The patient experienced circulatory collapse requiring intensive care unit (ICU) admission and high-dose norepinephrine six hours post-presentation, with concurrent positive anaerobic blood cultures prompting escalation to piperacillin-tazobactam. The ureteral stent was replaced the following day. Blood cultures revealed GPR on day 1, and* C. perfringens* was identified on day 2. However, urine culture results remained negative. The stent exchange proved effective, and the patient's condition stabilized without requiring drainage or surgical intervention. The hemolytic findings improved promptly following stent exchange. The patient was discharged from the ICU after six days and was transferred to another hospital 53 days later.

## Discussion

This case represents a healthcare-associated UTI caused by *C. perfringens*, with positive blood cultures but negative routine urine cultures and characteristic intravascular hemolysis. *C. perfringens* is an obligate anaerobic GPR found in the gastrointestinal and female genital tracts. In studies of patients with C. perfringens bacteremia, risk factors included age, malignancy, and diabetes mellitus [6], consistent with our case. *C. perfringens* bacteremia can be fatal, necessitating early diagnosis and treatment [1].

UTIs caused by *C. perfringens* are rare. While UTIs are rarely reported as a cause of *C. perfringens *bacteremia, they have been documented in healthcare settings [6]. However, the rarity of these UTIs may be attributable to frequent negative urine cultures for *C. perfringens*, as its obligate anaerobic nature precludes growth in routine cultures. In our case, although an initial urinary Gram staining showed GPR, the urine culture was negative. Early identification of GPR on urine Gram stainings aids diagnosis, but* Clostridium* species can rapidly decolorize during staining, appearing as Gram-variable or Gram-negative rods [2]. UTIs caused by GPR are uncommon and may be overlooked, delaying diagnosis [6]. The causative organisms in EPN are predominantly facultative anaerobic Gram-negative bacteria, such as *E. coli* and *Klebsiella* species, which can be detected through routine culture methods. However, in healthcare-associated EPN, obligate anaerobic bacteria such as *C. perfringens* must be considered, necessitating anaerobic culture techniques.

Intravascular hemolysis facilitates the diagnosis of *C. perfringens* bacteremia. *C. perfringens* is classified into types A-E based on its lethal toxins. All types produce alpha-toxin, which hydrolyzes red blood cell membrane phospholipids, causing intravascular hemolysis in approximately 10% of cases [3]. Peripheral blood smear tests reveal characteristic ghost cells due to hemoglobin loss [5]. Although not confirmed in our case, a rapid decrease in mean corpuscular volume due to acute red blood cell destruction is another key finding [5].

## Conclusions

This case demonstrates that *C. perfringens* can cause healthcare-associated UTIs, particularly EPN in high-risk patients with diabetes, malignancy, and recent urological interventions. The negative routine urine cultures despite active infection highlight the diagnostic challenge posed by this obligate anaerobic pathogen, emphasizing the importance of anaerobic culture techniques and careful Gram staining interpretation.

Clinicians should consider anaerobic pathogens in urinary tract infections with unusual presentations, particularly when intravascular hemolysis is present. Early recognition of characteristic findings, including dark red urine, GPR on urinary Gram staining, and hemolysis, can facilitate timely diagnosis and appropriate antimicrobial therapy, ultimately improving patient outcomes in these potentially life-threatening infections.
